# Evaluating different breast tumor progression models using screening data

**DOI:** 10.1186/s12885-018-4130-2

**Published:** 2018-02-20

**Authors:** Åsbjørn Schumacher Westvik, Harald Weedon-Fekjær, Jan Mæhlen, Knut Liestøl

**Affiliations:** 1 Department of Pathology, Oslo University Hospital, Norway, P.O. Box 4956 Nydalen, 0424 Oslo, Norway; 2 Oslo Center for biostatistics and epidemiology, Research Support Services, Oslo University Hospital, Norway, P.O. Box 4956 Nydalen, 0424 Oslo, Norway; 3 Center of Cancer Biomedicine, Department of Informatics, University of Oslo, Norway, P.O. Box 1080 Blindern, 0316 Oslo, Norway

**Keywords:** Breast cancer, Mammography screening, Hormone replacement therapy, Simulation model, Over-diagnosis, Tumor regression

## Abstract

**Background:**

Mammography screening is used to detect breast cancer at an early treatable stage, reducing breast cancer mortality. Traditionally, breast cancer has been seen as a disease with only progressive lesions, and here we examine the validity of this assumption by testing if incidence levels after introducing mammography screening can be reproduced assuming only progressive tumors.

**Methods:**

Breast cancer incidence data 1990–2009 obtained from the initially screened Norwegian counties (Akershus, Oslo, Rogaland and Hordaland) was included, covering the time-period before, during and after the introduction of mammography screening. From 1996 women aged 50–69 were invited for biennial public screening. Using estimates of tumor growth and screening sensitivity based on pre-screening and prevalence screening data (1990–1998), we simulated incidence levels during the following period (1999–2009).

**Results:**

The simulated incidence levels during the period with repeated screenings were markedly below the observed levels. The results were robust to changes in model parameters. Adjusting for hormone replacement therapy use, we obtained levels closer to the observed levels. However, there was still a marked gap, and only by assuming some tumors that undergo regressive changes or enter a markedly less detectable state, was our model able to reproduce the observed incidence levels.

**Conclusions:**

Models with strictly progressive tumors are only able to partly explain the changes in incidence levels observed after screening introduction in the initially screened Norwegian counties. More complex explanations than a time shift in detection of future clinical cancers seem to be needed to reproduce the incidence trends, questioning the basis for many over-diagnosis calculations. As data are not randomized, similar studies in other populations are wanted to exclude effect of unknown confounders.

**Electronic supplementary material:**

The online version of this article (10.1186/s12885-018-4130-2) contains supplementary material, which is available to authorized users.

## Background

The aim of breast cancer screening is to detect cancers at an early treatable state to reduce mortality. In Norway the introduction of organized biennial mammography screening in the age group 50–69 years was associated with a reduced breast cancer mortality [1], but also a marked increase in the incidence of invasive breast cancer in women invited to participate in the screening program [2, 3]. The incidence increased from 175 per 100,000 in the five-year period prior to screening to 350 per 100,000 during the prevalence screening in 1996–1997, leveling out at 300 per 100,000 in the succeeding period. Similar high levels during repeated screenings have occurred during introduction of mammography screening in other countries [4, 5].

The high detection rates observed during screenings may be due to several combined reasons. At the prevalence screening, many slow-growing tumors are detected due to the long time period from screening detectable size to clinically detectable size. In addition, there will be an age-shift in the detection affecting all rounds of screening. Accumulation of experience and improvements in the screening methodology may further enhance the detection rates somewhat over time. The breast cancer incidence may also change due to causes not related to screening, such as exposure to postmenopausal hormone replacement therapy (HRT). Finally, high detection rates over time may reflect that mammography is associated with overdiagnosis, i.e. the diagnosis of tumors that will never cause clinical disease in the life time of the women.

Breast cancer has traditionally been seen as an exclusively progressive disease [6], but recent observations of high incidence at repeated screenings have questioned these findings [7]. Hence, we aim to test whether the observed pattern of incidence changes observed in Norway can be explained using current progressive growth models, adjusting for the changes in HRT use at population level. To address this issue, we have developed a simple simulation model. Data from the pre-screening period and the prevalence screening are used to estimate growth rates and screening sensitivity. The model then predicts incidences in the subsequent period that can be compared to the observed levels.

## Methods

### Data description

#### Area and time period studied

Our aim is to reproduce the effect of mammography on cancer incidence from the initial four Norwegian screening counties Akershus, Oslo, Rogaland and Hordaland (AORH). Prior to 1996 there was no organized screening, while women aged 50–69 were invited to biennial screening from 1996 onwards. We have here simulated the 20-year period 1990–2009.

#### Demograpics

The four above-mentioned counties (AORH) represent 40% of the Norwegian population. Since fertility and mortality rates only varies moderately across Norway, we used the available data for all of Norway to mimic the relevant cohorts of the AORH-population. Data on cohort size and total mortality were obtained from Statistics Norway [8] and includes women born 1915–1965. A sensitivity analysis on the demographics is given in Additional file 1: Figure S1.

#### Size of screening-detected tumors

Our simulations are based on data from the Norwegian Breast Cancer Screening Program (NBCSP) [9] on women 50–69 years of age reporting no earlier mammogram and with breast cancer reported between 1996 and 2002. Of all tumors detected in NBCSP, 92% had a size measurement, resulting in a dataset of 907 tumors available for analysis. The mean tumor diameter is 15.6 mm (standard deviation 10.6 mm). According to the NBCSP guidelines [10] size was measured microscopically.

#### Size of clinically detected tumors

We use a dataset from Oslo University Hospital, based on pathology reports from the years 1988–1994. We reviewed 482 reports of primary invasive breast cancer in the age group 50–69. Of these, 468 cases (97%) had tumor size measurements. Forty cases are thought to have been detected at non-diagnostic mammography and excluded; thus 428 tumors remain for analyses. The mean diameter is 23.0 mm (standard deviation 14.9 mm). The tumors were measured microscopically. We also carried out simulations based on a dataset from Haukeland University Hospital in Bergen for sensitivity analyses. (For sensitivity analyses and graphical illustrations of the size distributions, see Additional file 1: Figure S2 and Figures. S3-S5, Table S1).

#### Breast cancer incidence

In the model, we needed age-specific incidences of 2 mm breast cancer tumors to initiate the simulation of each tumor (which start at size 2 mm). To approximate this, we use age-specific incidences from the pre-screening period derived from the NORDCAN database [11] (Additional file 1: Figure S6). Since this data represent detected tumors, the curves are shifted towards lower ages until the age-specific incidence of detected tumors in the model approximately corresponds to the NORDCAN data (shifts of the order of two years).

#### Long-term trend in breast cancer incidence

The Norwegian incidence of breast cancer for women 50–69 years of age increased gradually during the period 1950–1995, and it appears reasonable that such an increase would have continued also in the absence of screening. Hence, we added a term to the incidence increasing linearly over time. Sensitivity analysis was carried out by testing both steeper and less steep age dependence of the incidence, and by eliminating the time trend (Additional file 1: Figures S7-S8).

#### Screening attendance

The attendance in the AORH-counties has been around 78%. For our main model we assume that every woman has a 78% chance of attending any round of mammography, regardless of her earlier attendance. Alternatively, Additional file 1: Figure S9 shows results where each woman either attends all or no screenings.

#### Hormone-replacement therapy adjusted incidences

Hormone treatment is known to increase breast cancer risk [12, 13], and Norway experienced a large increase in use just around the start of public screening [14]. Hence, hormone treatment use should be accounted for when studying breast cancer progression. As the increased breast cancer risk is dependent on the type of hormone treatment, length of use and starting age [13, 15], we base our hormone treatment correction on estimates from Norwegian hormone treatment use. Both Bakken et al. [15], and Weedon-Fekjær et al. [14], estimated between 2.1 and 2.2 increased relative risk of active hormone treatment users in Norway. In order to derive breast cancer incidence trends which are adjusted for hormone treatment, we utilized data on Norwegian hormone treatment sales. We applied a one year lagged 2.2 relative risk of hormone treatment use on Norwegian sales figures, which enabled us to calculate estimated breast cancer incidence trends (adjusted for HRT) [14]. As the estimated relative risk of hormone treatment, and the estimated lag from use to increased risk is based on much of the same data and user patterns as this study, the estimate should be relatively robust for derivations of user patterns or type of hormone treatment applied.

### Estimation of growth rates and screening sensitivity

The growth estimates from Weedon-Fekjær et al. [16] were updated applying our clinical dataset. Separate growth rate distributions were also obtained with and without the HRT-adjusted effect. For robustness analyses we ran simulations with our alternative clinical dataset as well as other growth rates found in the literature [16, 17] (Additional file 1: Table S2-S3). Screening sensitivity as a function of tumor size was described by a logistic curve. We estimated the two parameters describing the location and slope of the curve by optimizing the fit to the observed data using grid search. For given parameter sets, simulated women were generated according to our model and we registered the size of tumors detected at a mimicked prevalence screening. The fit between the simulated and observed tumor size distribution was scored by a least square criterion, and the best fitting parameter set was used (Additional file 1: Table S4). For the final model the sample size of tumors generated was set to 1,000,000.

### Simulation model description

The intention behind our simulations is to obtain age-specific breast cancer incidence curves for the years 1990–2009 based on chosen sets of parameters determining tumor growth and detection. The results are based on creating large samples of simulated women followed from birth to age 75. For the final main simulated incidence curve results, the sample size is 100,000,000, implying that random error is negligible compared to the variations found for the natural population in the AORH-counties.

The simulation model was written in Java SE 7 [18], using the standard random number procedures of the platform. When drawing according to age- or time-dependent curves, piecewise linear approximations were applied so that the inversion principle could be used to obtain random variates closely approximating the desired distributions.

Women are simulated according to the following scheme:

- Each woman gets a year of birth and a potential life duration based on stochastic drawings according to the demographic data described in the previous section.
- Stochastic drawings according to the cohort and age dependent probabilities determine if and at which age the women get breast cancer.
- Tumors grow according to a monotonous growth model. The form of the growth curve as well as the assumed maximal tumor size was based on earlier models used to mimic observed growth patterns [17] (the exact choice of growth curve have been reported to be of limited importance, see Weedon-Fekjaer et al. [16]). We then used a generalized logistic growth curve with tumor diameter at time t given as:
  $$ S(t)={S}_{\infty }{\left(1+{e}^{-N\left( bt+c\right)}\right)}^{-\frac{1}{3N}} $$

where S∞ is the assumed maximum tumor diameter of 128 mm, b the tumor growth rate, *N* = ¼, and c is chosen so that size = 2 mm at time zero.

Parameter b is drawn from a log-normal distribution. In our main simulation where we have added the HRT-adjusted effect, the distribution has parameters with a mean of 0.70 and spread of 1.30 for a model with time t given in months. Parameters used for the other simulations can be found in Additional file 1: Table S2-S3.

- For each tumor, a clinical detection size was determined using random drawings from the empirical size distribution. Based on this size and the growth curve obtained in the previous step, the time of (potential) clinical detection is calculated. If the tumor has not been detected at mammography (see below), this case is registered as a clinical detection at the given time and size.
- During the eligible age-interval the woman is “invited” to biennial mammography screening, and attends with the empirically observed attendance rate of 78%. If a woman with breast cancer attends, a drawing with probability obtained from the logistic sensitivity curve determines if the tumor is detected.

As a second step in our modeling we added non-progressive tumors. One set of simulations were run with the addition of indolent tumors, which we defined as tumors that stop growing at a sub-clinical size, but remain present and may be detected by mammography. Another group of non-progressive tumors we introduced separately were tumors that grow similar to limited malignant potential tumors (LMP-tumors) introduced by Fryback et al. [19]. The tumors had the following characteristics:

- The tumors initially grow according to the same growth rate distribution as defined above for the regular tumors.
- An individual maximum size is drawn from a log-normal function (for the standard LMP-tumors mean = 2.5, spread = 0.36).
- The LMP-tumor is at its maximum size for 12 months before entering a non-detectable state, while dormant tumors remain stable in size.

**Fig. 1:**
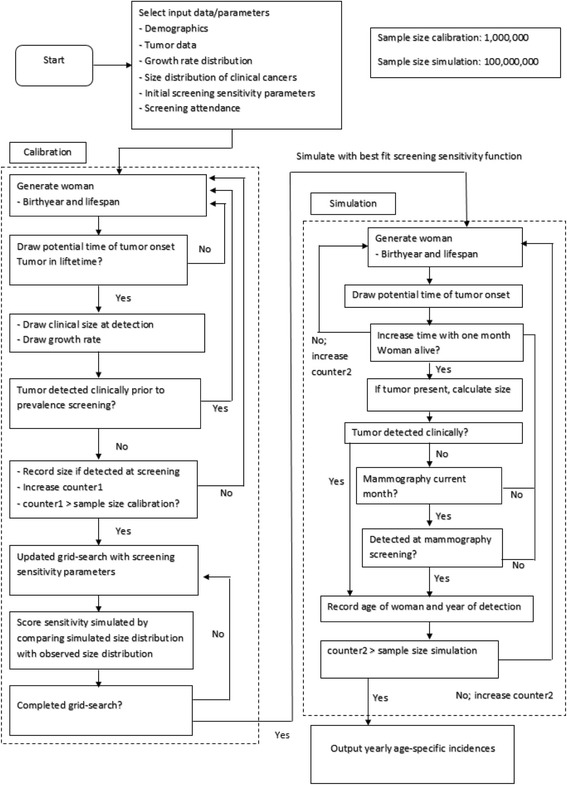
Flow-chart of the simulation model

Figure 1 shows a flow-chart of the simulation model.

## Results

### The empirically observed incidence

**Fig. 2:**
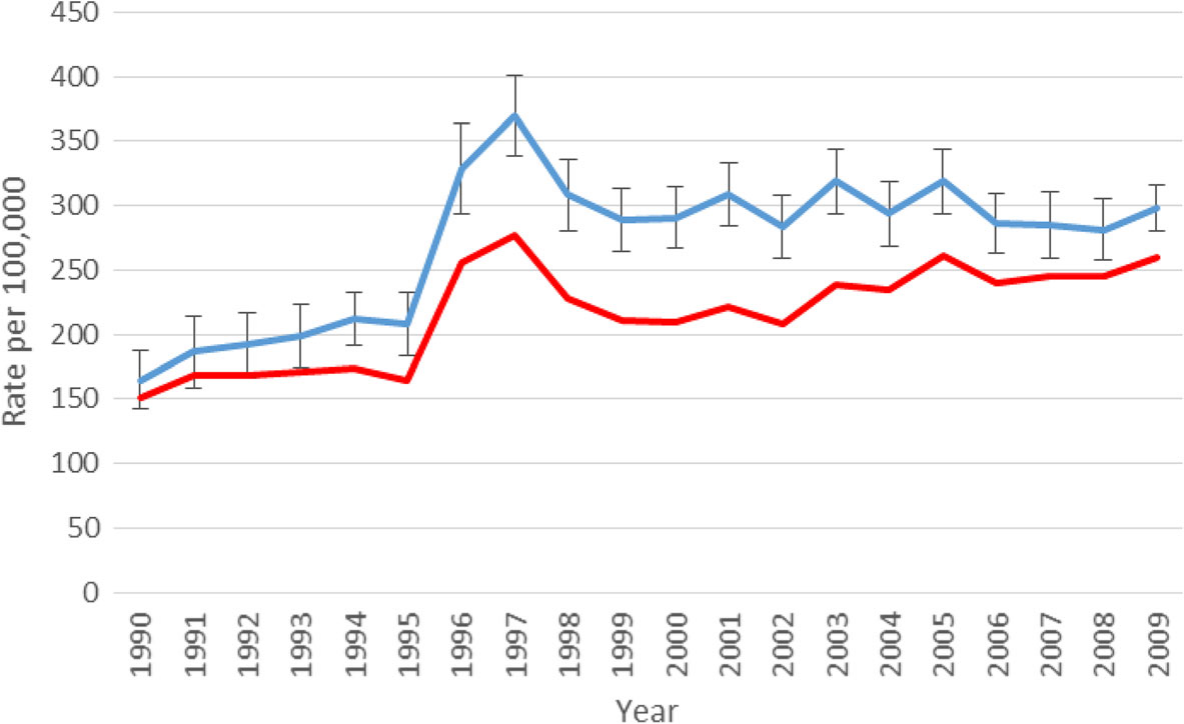
Observed (blue) and HRT-adjusted (red) incidences in the AORH-counties for women aged 50–69, with 95% pointwise confidence interval bars added to the observed data

Figure 2 shows the 1990–2009 breast cancer incidence trend for women 50–69 years of age. Prior to 1995/1996 only opportunistic mammography took place, with a moderate increase in the observed incidence and about 200 new breast cancer cases per 100,000 women. The incidence then rose sharply to 350 cases per 100,000 women in the years of the prevalence screening, and leveled out at about 300 cases per 100,000 women in the following years (Fig. 2, blue curve). The red curve in Fig. 2 is based on assessment of the effect of HRT, and shows estimates of the counterfactual incidence curve in the absence of artificial hormones use. This adjustment for HRT use results in a less steep increase in incidence prior to 1996, and lower estimates throughout the screening period.

After the introduction of screening, the incidence in women age 70 and up has stabilized at a level slightly below the pre-screening (Additional file 1: Figures S10-S11).

**Fig. 3:**
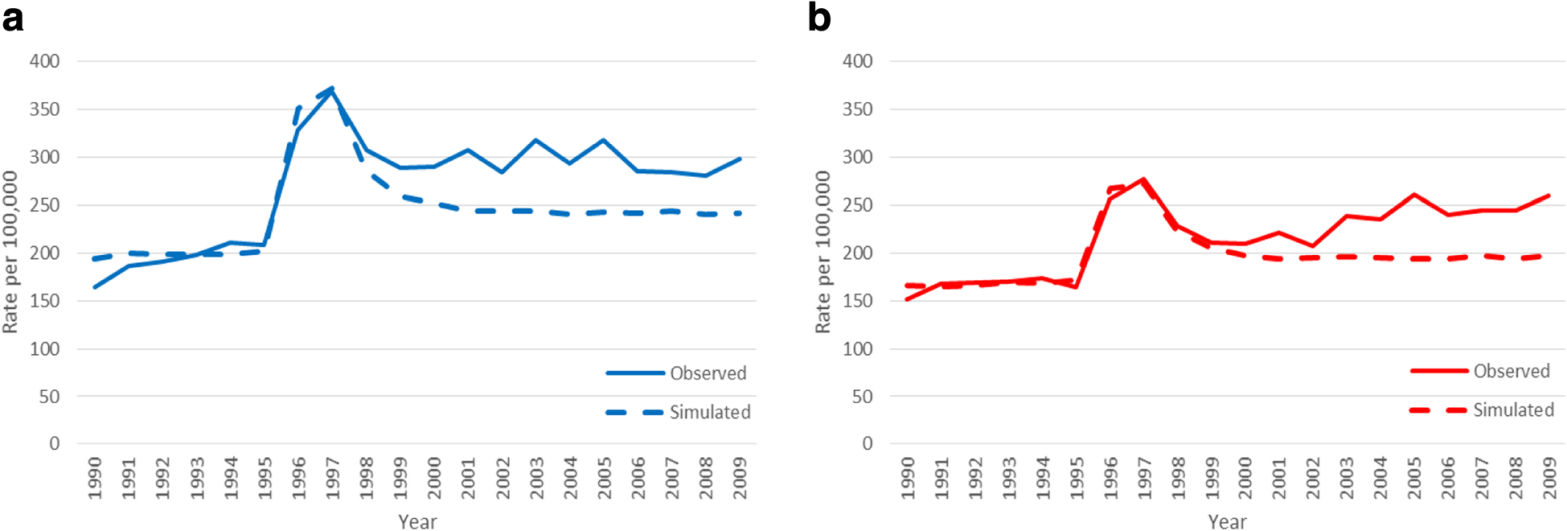
Comparisons of observed data (solid lines) and model estimates (dotted lines). **a**) Observed incidence **b**) HRT-adjusted incidence

Figure 3a and b compares model output and observed levels for unadjusted and HRT-adjusted incidences, respectively. As expected, the model reproduces the incidence prior to screening introduction and the prevalence peak, for both the un-adjusted and the HRT-adjusted incidence. However, when simulating past the prevalence round and into subsequent rounds of screening, the model obtains an incidence level that is clearly below what is observed. At the end of the simulation period when HRT use was moderate, the observed incidence is of the order of 25% above the simulated level both in the unadjusted and HRT-adjusted scenarios.

As illustrated in various sections of the Additional file 1, moderate changes in demographics, the age-specific cancer incidences, the observed pre-screening dataset or the model for clinical detection of cancers do not alter the qualitative aspects of the results.

**Fig. 4:**
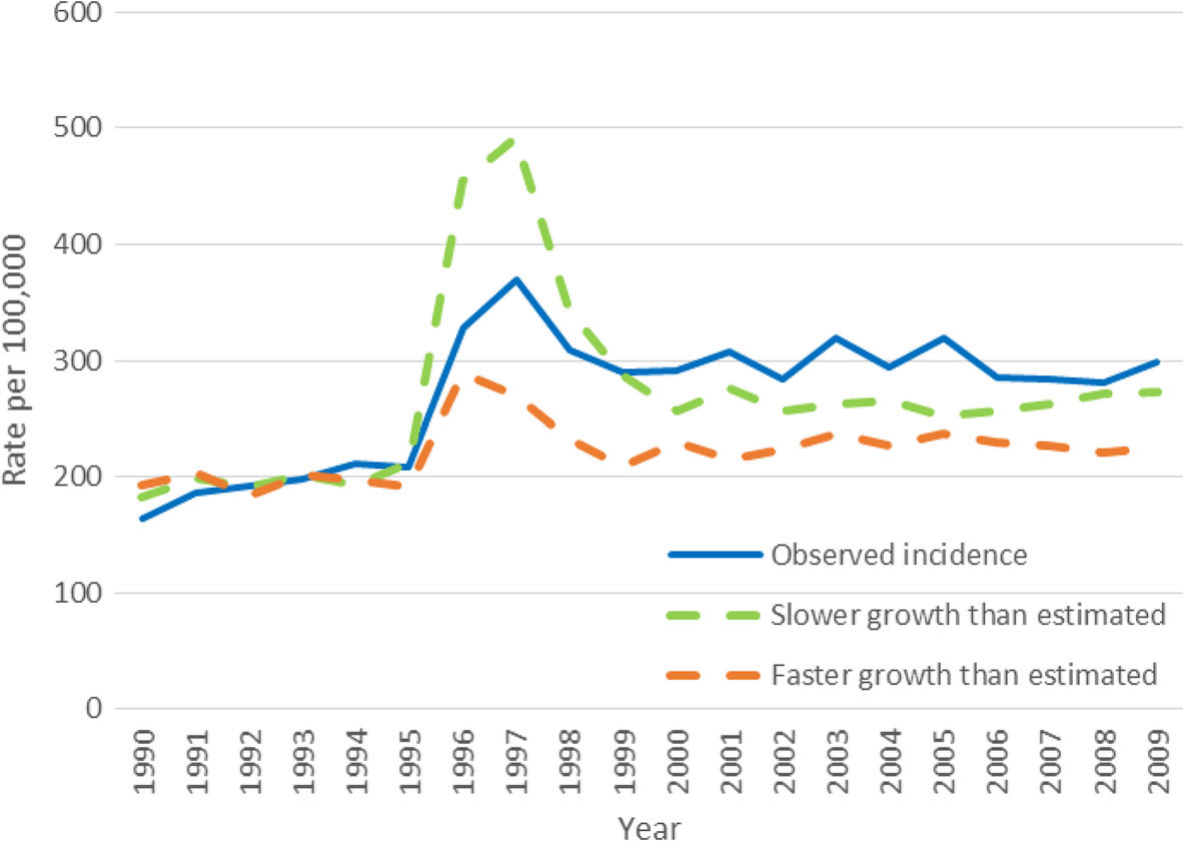
Illustration of effects of marked changes in growth estimates; Solid blue line = observed incidence, orange curve = faster growth than earlier estimated, green curve = slower growth than earlier estimated

To test the effect of drastic changes in growth parameters, we performed simulations with growth estimates far away from the estimated values. None of the simulations with monotonous growth estimates resulted in estimates approximating the observed incidence patterns (Fig. 4).

Our simulations with non-progressive indolent tumors resulted in more tumors being detected after the introduction of mammography screening. We were, however, not able to fit all aspects of the observed incidences. There was a tradeoff between fitting the prevalence peak and the subsequent levels (Additional file 1: Figure S12), that is, the prevalence peak tended to be too high and the subsequent level too low.

**Fig. 5:**
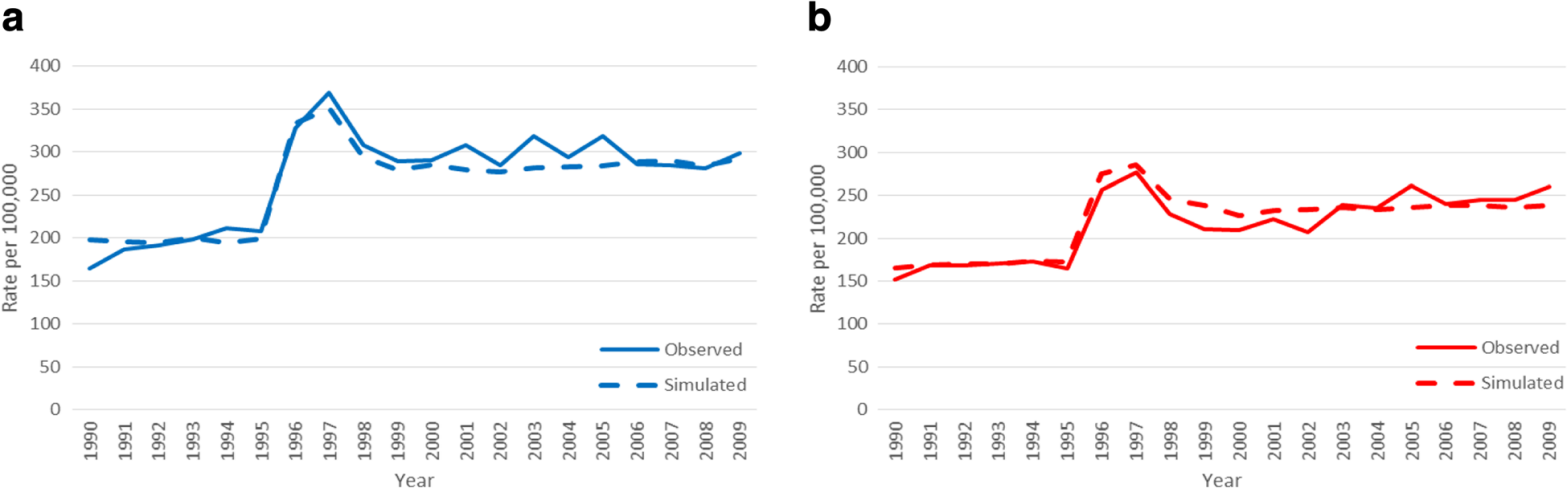
Simulated (dotted line) and observed (solid line) incidence when introducing a group of tumors which are not detectable on repeated screening examinations: **a**) observed incidence **b**) estimated incidence adjusted for HRT use

After introducing tumors of the LMP type, that stop growing at a sub-clinical size and later become undetectable, we were able to approximate both the prevalence peak and the level at subsequent rounds of screening (Fig. 5).

## Discussion

We aimed at reproducing the observed breast cancer incidence trend in the AORH-counties in Norway after the introduction of organized mammography screening based on the classic progressive tumor growth assumption. The simulated incidence levels were markedly below the observed levels for the subsequent rounds of screening. The results were robust to changes in most parameters such as screening attendance, demographics and size distributions of clinical detected cancers.

The effects of mammography screening on cancer incidence have been studied since the 1970s, with diverging results [1, 20–27]. One key factor for the effect of mammography screening on cancer incidence is breast cancer tumor growth and development. The growth and development of breast cancer has been studied using both analytic [16, 17] and simulation approaches [28, 29]. In the CISNET collaboration [29], collaborative modelling have been used to investigated many aspects of mammography screening including mortality reduction, treatment effects and overdiagnosis. All breast cancer simulation models are, however, based on some set of assumptions that will influence the results of the model. In the CISNET collaboration, several different models have been applied to show the effect of different model assumptions. We developed a model partly inspired by the CISNET simulation models [29–31], and combined it with estimates of tumor growth and screening sensitivity based on pre-screening and prevalence-screening data. As the results show, we are unable to reproduce the observed data without introducing so-called LMP-tumors, which were also used by the Wisconsin CISNET group [19] to explain the trends in their US data. On the other side, several CISNET models have shown decent fit using US data simulating only progressive tumors [32, 33]. Our model is, however, based on much more detailed screening data than US CISNET breast cancer modelling.

For an estimate including use of both progesterone-estrogens combinations and pure estrogen, our 2.2 relative breast cancer risk is relatively high [12, 13, 34] compared to earlier studies, as pure estrogens use probably have less effect on breast cancer risk than progesterone-estrogens use. Hence, we could be fairly certain that at least the majority of the potential increased risk due to hormone treatment use is accounted for in our hormone treatment adjusted rate.

Following the Wisconsin-model [19] we introduced so called LMP-tumors that typically stop growing at a sub-clinical size and later become undetectable. The LMPs are modeled in a simple way and without confirmed biological parallels. However, the simulations indicate that the existence of tumors that reach a stage where they are detectable on a mammogram, but subsequently undergo changes that make them markedly less detectable, can help explain the observed incidence pattern.

The datasets on tumor sizes are essential to the modeling. However, some tumors in the datasets lack a size measurement; 8% of the screen-detected tumors and 3% of the clinically detected tumors. When looking at the information concerning screen-detected tumors lacking size, most have a classification code stating the tumors have grown into skin and/or chest wall, hence measuring a diameter is impossible. Moreover, although we have tried to remove cases detected at non-diagnostic mammography from the clinical dataset, some such cases may remain. Thus the sizes of both clinical and screen-detected tumors may be underestimated. On the other hand, the average size reported from four randomized trials is about as the same as for our data (mean 21 mm in the non-mammography arm, 16 mm among screened tumors [21]). Furthermore, although there may be some systematic errors in the size measurements, the sensitivity analyses using the alternative clinical dataset with larger measured tumors resulted in similar qualitative results (Additional file 1: Figure S2).

A strength in our model is the high-quality data from the Norwegian Cancer Registry [35]. The reporting of cancer to the Norwegian cancer registry is mandatory, and diagnostic information is obtained separately from clinicians, pathologists, and death certificates, with only 0.2% of all cancers ascertained only from death certificates. The Registry is also responsible for NBCSP, and the introduction of screening in Norway occurred in a systematic fashion with county wise introduction of mammography and systematic collection of data, including data on tumor size and questionnaire data on earlier mammography.

Our model aims at simulating the female population in the AORH counties. Some of the data are, however, based on statistics from all of Norway. Comparisons of results from mimicking the real population and using uniform numbers born each year strongly indicate that small or moderate changes in demographics are of limited importance. The results are also robust to moderate changes in underlying incidence trends.

Opportunistic mammography poses specific challenges when modeling the incidence trends. Estimates based on information obtained in the NBCSP program as well as in The Norwegian Women and Cancer Study indicate fairly high levels of mammography prior to the organized screening [36]. Given these estimates, the empirically observed increase in incidence seen prior to 1996 is surprisingly low, possibly indicating low sensitivity of the unorganized screening. If one believes there is a large effect of opportunistic mammography this would mainly affect the prevalence peak, making it even harder to reproduce the observed data, see Additional file 1: Figure S13.

The available estimates on tumor growth are not vastly different despite being based on entirely different types of data [16, 17]. Still, robustness tests of the model are important. Lower growth rates would in the presence of strictly progressive tumors not only increase the observed incident during subsequent screening, but also the level on initial screening (Fig. 4). Generally, our tests of various growth rates indicate that variations in such rates cannot fully explain our missing fit when assuming only progressive tumors.

The use of HRT increased dramatically in Norway from the mid-90ies, about simultaneously as the introduction of organized screening, and remained high until a sharp decrease from around 2002 [37]. The coincidence of the increase in the use of hormones and the introduction of screening poses a challenge when interpreting the data. We did a separate set of simulations based on incidence rates adjusted for the effect of HRT. Our main conclusions remained unchanged, we are able to reproduce the background incidence and the prevalence peak, but unable to reproduce the subsequent level. The gap is smaller, indicating that HRT is a likely contributor to the higher observed level in subsequent screening rounds, but the gap is still substantial especially from around 2005.

Another factor possibly contributing to increasing incidence rates is the introduction of improved screening techniques, including the introduction of digital mammography. However, the fact that the size of the detected tumors have not decreased significantly over time [38] indicate a moderate effect since a marked effect is necessary to maintain a high incidence level over a long time period.

The introduction of mammography has led to a substantial increase in the detection and surgical removal of ductal carcinoma in situ (DCIS) [39]. The justification for treating DCIS is that some cases will develop into invasive cancer if left untreated [40]. Early treatment of DCIS should therefore reduce the future incidence of invasive cancers. Consequently, it is even harder to explain the incidence during subsequent screening if a substantial number of screening detected DCIS cases would have progressed to invasive cancers if left untreated [28].

Introducing tumors that regress, or enter an undetectable state, enable us to approximate observed incidence rates. Regressing tumors are known for certain cancer types, including neuroblastoma [41], but is regarded as uncommon for most cancer types. There are some case reports of likely clinical spontaneous regression of breast tumors [42, 43], but since cases are confirmed through surgery or biopsy, interfering with the biology, there are few good possibilities to firmly observed spontaneous regression clinically.

One possible explanation for regressive changes being more common in breast cancer might be the dependence on growth factors, particularly estrogens. About 70% of breast tumors are reported to carry estrogen receptors [44]. The hormone levels drop dramatically around menopause, followed by a slow further decline [45]. The age group invited to screening overlaps the population undergoing menopause. Thus, it seems likely that many tumors will encounter a decreasing growth stimulus, potentially resulting in slower growth rates, and maybe opening for the possibility of regressive changes. The effect of hormones on breast tumor growth is also supported by the shape of the age-incidence curve [46], the effect of anti-estrogen drugs on relapse of breast cancers [47] and the effect of HRT [48]. It is also conceivable that the occurrence of regressive changes is increased in periods with frequent use of external hormones, due to hormone levels being increased for a period of use with a marked drop in hormone levels at HRT termination. There are, however, no direct biological observations of tumor regression as a consequence of natural changes in hormone levels. One may also argue that the changes in hormone levels after 60 years of age are too moderate to be a likely cause of regression. Existence of such tumor changes would, however, give a possible explanation of the high incidence during repeated screenings and should be studied further.

Other model-based studies have also pointed at regressive tumor changes as possible explanations for trends seen in the considered data. As earlier stated, Fryback et al. [19], in their Wisconsin CISNET model of incidence trends, concluding that high numbers of LMP-tumors were necessary to obtain satisfactory fits to their data. Moreover, Zahl et al. reached similar conclusions in two studies comparing cohorts with repeated screenings over a 6 years period to cohorts with only one screening at the end of the 6 years period [2, 7].

Breast cancer risk is known to decline when women leave mammography screening programs [14], but the key question regarding mammography screening overdiagnosis is to what degree this decline eradicates the earlier increase in breast cancer incidence during mammography screening. Working with strictly progressive natural history models, breast cancer overdiagnosis will always be low for any mammography screening with substantial life expectancy beyond the last screening exam [49]. On the other hand, there are many epidemiological studies indicating substantial levels of overdiagnosis [50]. If some tumors are not strictly progressive, as indicated in this study, future overdiagnosis estimates should be made with great care not fixing the study to assumptions of only progressive breast cancer tumors.

To summarize, the observed trends in Norwegian breast cancer incidence around screening introduction is not possible to reproduce by simulating data with only strictly progressive breast cancer tumors. There is hence indications that some screening detected tumors might had regressed to a non-screening detectable phase in the absence of screening detection. Epidemiological data have, however, many potential pitfalls and there is great need of more studies looking into breast cancer incidence around screening introduction, based on data from other countries with good cancer registries.

## Conclusion

The observed change in incidence around the introduction of screening is hard to reproduce using documented growth rate estimates, opening the possibility for regressive changes in some cancers making them less detectable at repeated mammograms.

## Additional file

Additional file 1: **Figure S1.** Comparison of results based on static population. **Figure S2.** Results of running simulation on basic model using clinical dataset from Oslo University Hospital (blue) versus clinical dataset from Haukeland University Hospital in Bergen (green. **Figure S3.** Clinically detected cancers, A = Haukeland University Hospital, B = Oslo University Hospital, Ullevål. **Figure S4.** Prevalence screening detected cancers. Size distribution. **Figure S5.** Comparison of the size distributions of tumors detected at the prevalence screening (red) and in pre-screening years (data from Oslo University Hospital in blue, from Haukeland University Hospital in green). **Figure S6.** Data from NordCan, with historic incidence-data for various cohorts at specific ages. **Figure S7.** Expected and observed incidences in the age groups 50–69 applying different rates of increased incidence over time. **Figure S8.** Incidences in the age groups 50–69 where we have varied the probability of getting a 1 mm cancer depending on age. **Figure S9.** Comparison of results when simulating different screening attendance scenarios. **Figure S10.** Observed female breast cancer incidence in Norway from 1953 to 2013. **Figure S11.** Observed incidence in the AORH-counties from 1990 to 2009. **Figure S12.** Simulation results with dormant tumors which are not detected clinically. **Figure S13.** Effect of adding two hypothetical rounds of opportunistic screening (1992–1993 and 1994–1995). **Table S1.** Table of parameters used in log-normal function to reproduce Fig. 2 in the main article. **Table S2.** Time in years it takes for a tumor to advance from 10 mm to 20 mm using different growth estimates. **Table S3.** The parameters used for the lognormal growth distributions. **Table S4.** Parameters of the logistic sensitivity functions after calibration (parameters are in the format [mean,spread]). (DOCX 458 kb)

## Abbreviations

AORH: Akershus, Oslo, Rogaland and Hordaland
DCIS: Ductal carcinoma in situ
HRT: Hormone replacement therapy
LMP: Limited malignant potential
NBCSP: Norwegian Breast Cancer Screening Program
