# Supplementary material for: Evaluating different breast tumor progression models using screening data
Source: BMC Cancer. 2018 Feb 20;18:209. doi: 10.1186/s12885-018-4130-2 (PMC5819671; doi:10.1186/s12885-018-4130-2)
Supplement: Supplementary file 1 — Figure S1. Comparison of results based on static population. Figure S2. Results of running simulation on basic model using clinical dataset from Oslo University Hospital (blue) versus clinical dataset from Haukeland University Hospital in Bergen (green. Figure S3. Clinically detected cancers, A = Haukeland University Hospital, B = Oslo University Hospital, Ullevål. Figure S4. Prevalence screening detected cancers. Size distribution. Figure S5. Comparison of the size distributions of tumors detected at the prevalence screening (red) and in pre-screening years (data from Oslo University Hospital in blue, from Haukeland University Hospital in green). Figure S6. Data from NordCan, with historic incidence-data for various cohorts at specific ages. Figure S7. Expected and observed incidences in the age groups 50–69 applying different rates of increased incidence over time. Figure S8. Incidences in the age groups 50–69 where we have varied the probability of getting a 1 mm cancer depending on age. Figure S9. Comparison of results when simulating different screening attendance scenarios. Figure S10. Observed female breast cancer incidence in Norway from 1953 to 2013. Figure S11. Observed incidence in the AORH-counties from 1990 to 2009. Figure S12. Simulation results with dormant tumors which are not detected clinically. Figure S13. Effect of adding two hypothetical rounds of opportunistic screening (1992–1993 and 1994–1995). Table S1. Table of parameters used in log-normal function to reproduce Fig. 2 in the main article. Table S2. Time in years it takes for a tumor to advance from 10 mm to 20 mm using different growth estimates. Table S3. The parameters used for the lognormal growth distributions. Table S4. Parameters of the logistic sensitivity functions after calibration (parameters are in the format [mean,spread]). (DOCX 458 kb) [file 12885_2018_4130_MOESM1_ESM.docx]

**SUPPLEMENTARY**

1. **Demographics**


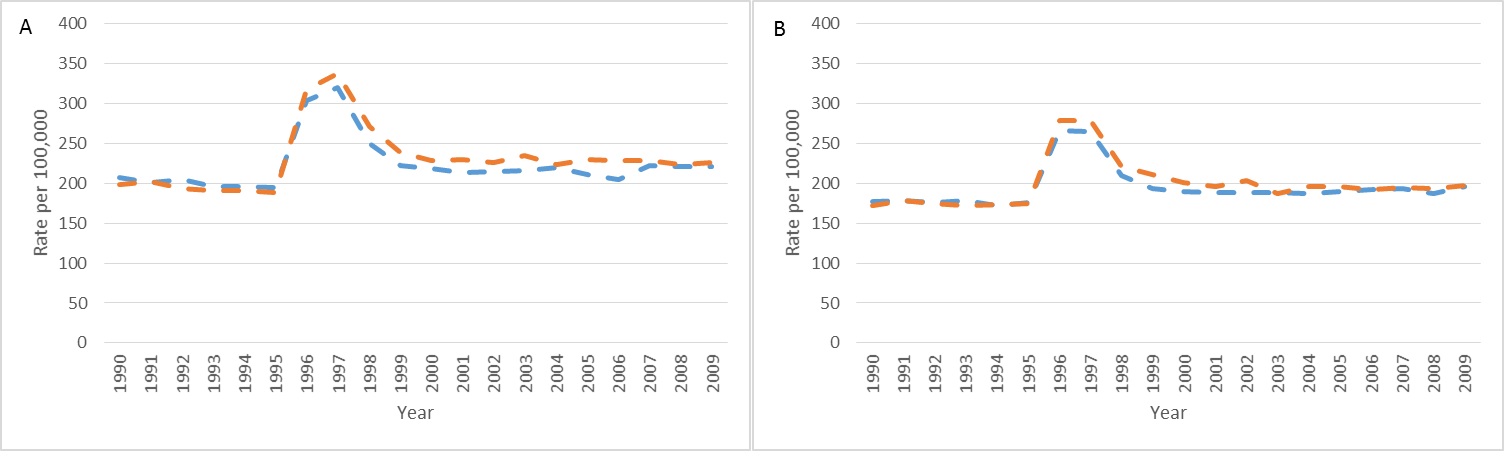


Figure S1: Comparison of results based on static population (orange), with equal number of births each year, and actual population (blue) on unadjusted incidence (A) and HRT-adjusted incidence (B).

1. **Clinical Dataset Sensitivity Analyses**


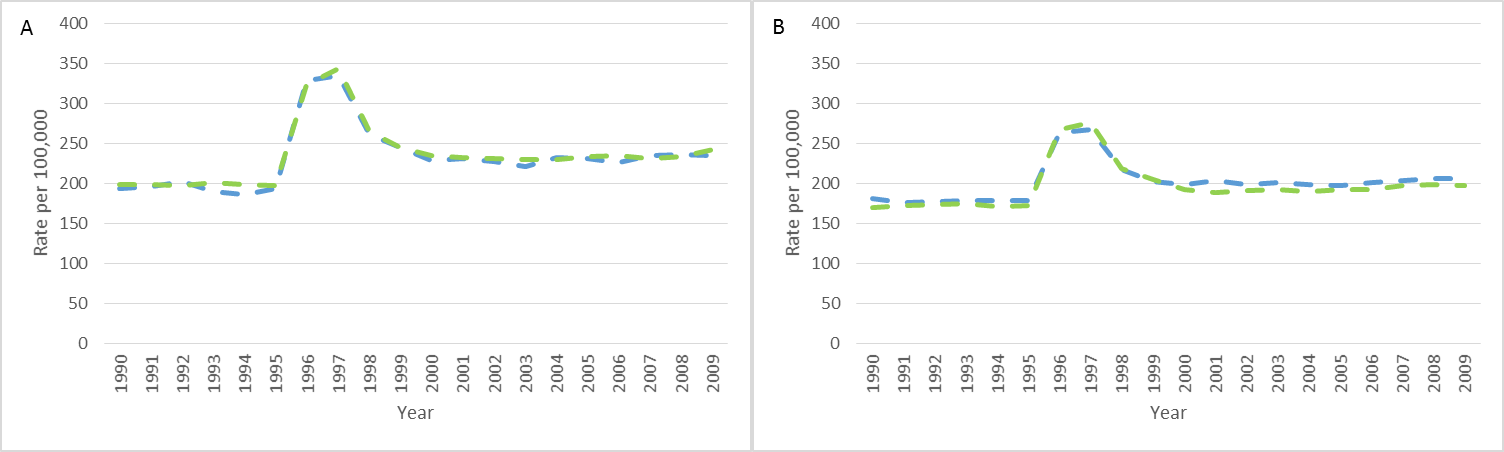


Figure S2: Results of running simulation on basic model using clinical dataset from Oslo University Hospital (blue) versus clinical dataset from Haukeland University Hospital in Bergen (green). A = unadjusted incidence, B = HRT-adjusted incidence.

1. **Raw Input Datasets**


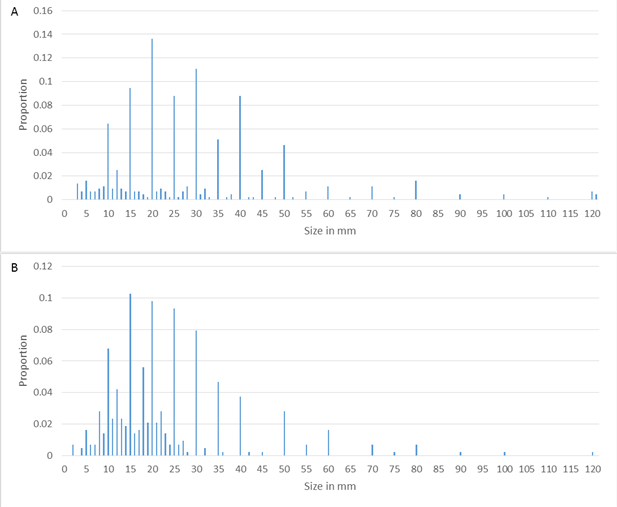


Figure S3: Clinically detected cancers, A = Haukeland University Hospital, B = Oslo University Hospital, Ullevål.


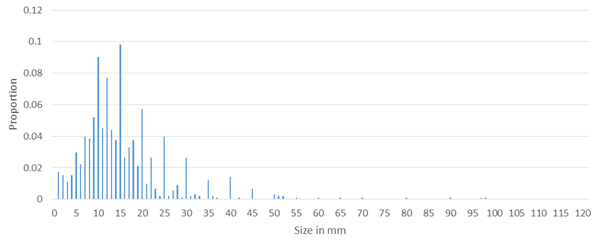

Figure S4: Prevalence screening detected cancers. Size distribution.


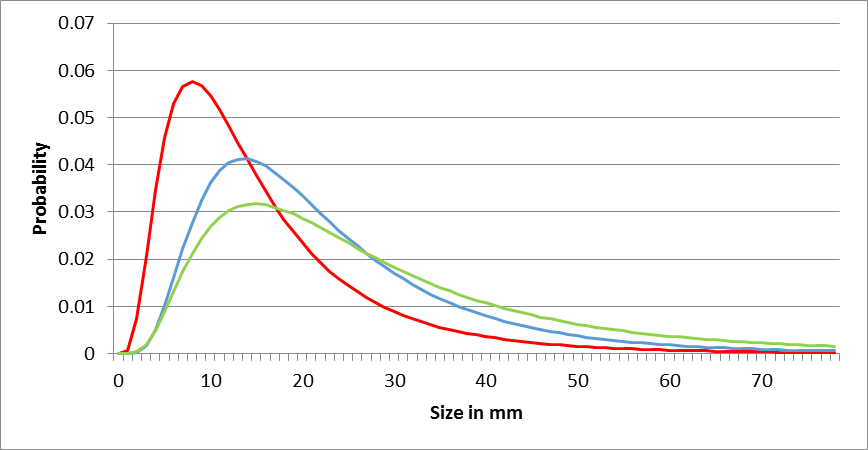


Figure S5: Comparison of the size distributions of tumors detected at the prevalence screening (red) and in pre-screening years (data from Oslo University Hospital in blue, from Haukeland University Hospital in green). Curves are obtained by fitting log-normal distributions to the data.

|  | µ | Σ |
| --- | --- | --- |
| Haukeland | 3.150 | 0.678 |
| Ullevål | 2.964 | 0.596 |
| Prevalence Screening | 2.546 | 0.685 |

Table S1: Table of parameters used in log-normal function to reproduce Figure 2 in the main article.

1. **NORDCAN data, used to set background incidence**


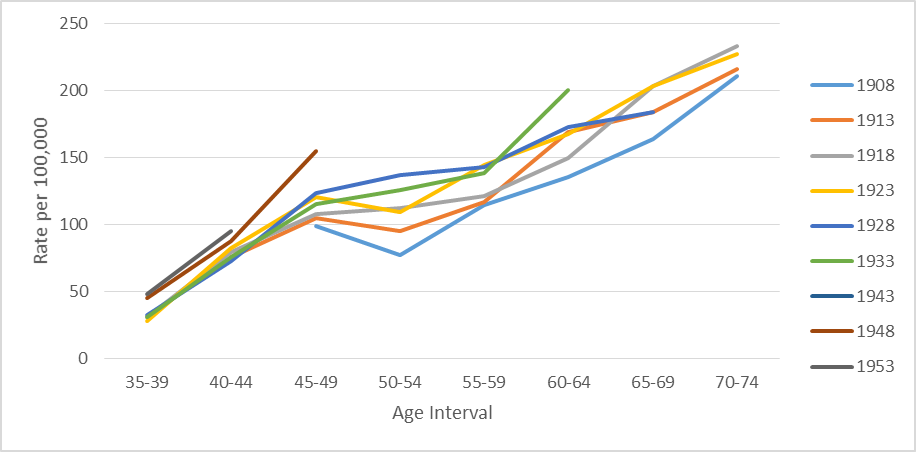


Figure S6: Data from NordCan, with historic incidence-data for various cohorts at specific ages.

1. **Effect of trend in incidence.**


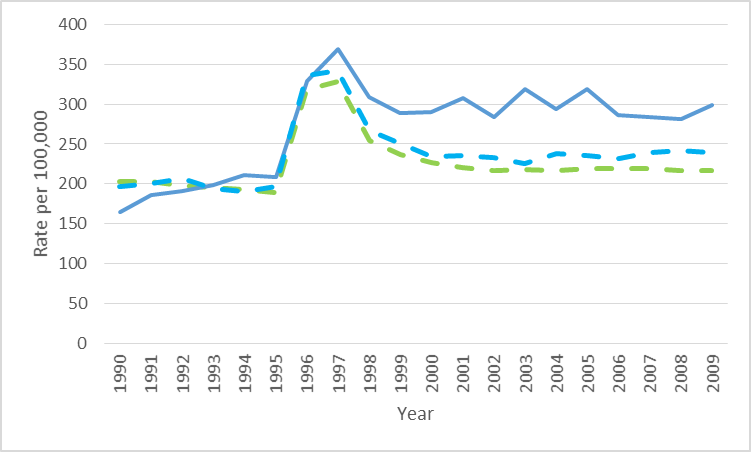


Figure S7: Expected and observed incidences in the age groups 50-69 applying different rates of increased incidence over time; Green dashed = no increase of incidence over time, Blue dashed = moderate increase incidence over time, Blue solid = observed incidence.


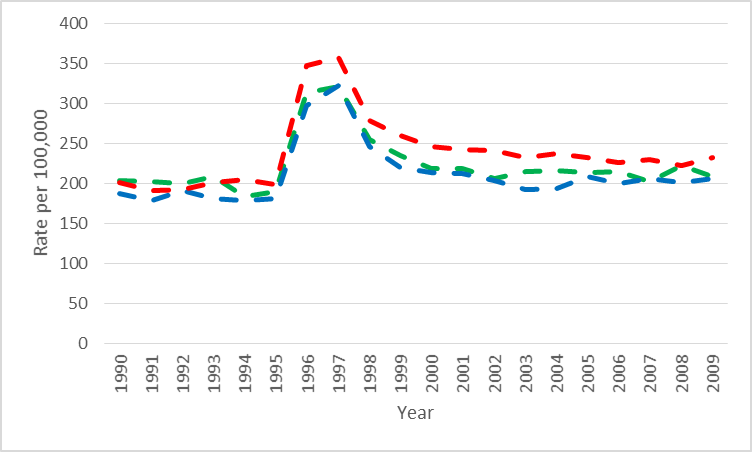


Figure S8: Incidences in the age groups 50-69 where we have varied the probability of getting a 1 mm cancer depending on age. The proportion of cancers initiated is the same in all 3 curves. Blue = original, green = more cancers in the older women, red = more cancers in the young women.

1. **Effect of screening attendance.**


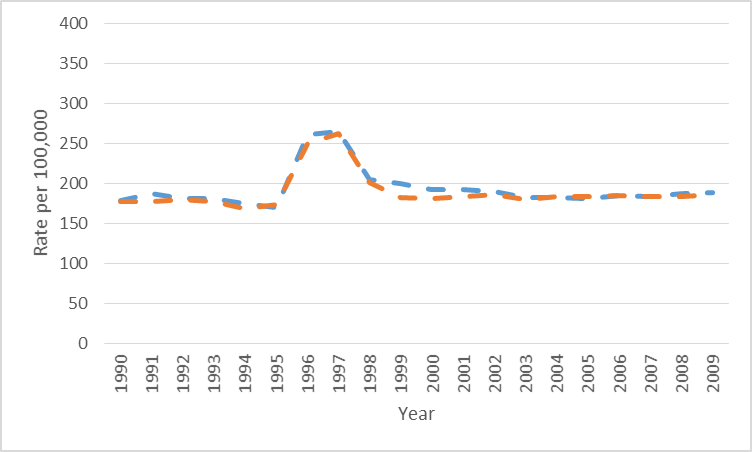


Figure S9: Comparison of results when simulating different screening attendance scenarios; one where 78 % of women attend all rounds of screening and 22% never attends (orange), and one where a woman has a 78 % chance of attending any screening independent of earlier attendance (blue). Simulations are run with the HRT-adjusted growth estimates.

**G. Growth estimates**

| Percentile | Spratt [17] | Weedon-Fekjær [16] | Generalized logistic Haukeland unadjusted (simulated) | Generalized logistic Haukeland HRT-adjusted (simulated) | Generalized logistic Ullevål unadjusted (simulated) | Generalized logistic Ullevål HRT-adjusted (simulated) |
| --- | --- | --- | --- | --- | --- | --- |
| 1 (least agressive) | 12.9 | 10.9 | 25.9 | 12.7 | 47.58 | 18.19 |
| 5 | 4.6 | 6.3 | 9.92 | 5.82 | 18.24 | 8.37 |
| 25 | 1.5 | 2.0 | 2.54 | 1.92 | 4.65 | 2.76 |
| 50 | 0.8 | 0.9 | 0.983 | 0.892 | 1.80 | 1.28 |
| 75 | 0.5 | 0.4 | 0.383 | 0.408 | 0.692 | 0.60 |
| 95 | 0.2 | 0.1 | 0.10 | 0.133 | 0.183 | 0.192 |
| 99 (most agressive) | 0.1 | 0.0 | 0.033 | 0.058 | 0.067 | 0.092 |

Table S2: Time in years it takes for a tumor to advance from 10 mm to 20 mm using different growth estimates.

| **Simulation** | **Mean** | **Spread** |
| --- | --- | --- |
| Haukeland unadjusted incidence | 0.967 | 1.98 |
| Haukeland HRT-adjusted incidence | 1.07 | 1.31 |
| Ullevål unadjusted incidence | 0.36 | 1.98 |
| Ullevål HRT-adjusted incidence | 0.70 | 1.30 |

Table S3: The parameters used for the lognormal growth distributions

1. **Screening Sensitivity**

|  | Haukeland dataset | Ullevål dataset |
| --- | --- | --- |
| Unadjusted growth estimate | 1.18, 7.54 | 1.34, 9.0 |
| HRT-adjusted growth estimates | 1.21, 7.61 | 1.36, 9.2 |

Table S4: Parameters of the logistic sensitivity functions after calibration (parameters are in the format [mean,spread]). The logistic sensitivity function is used when a woman attends screening. Using the size of the tumor at the time of screening, the tumor is detected with a probability defined by the sensitivity function.

1. **Observed incidences**


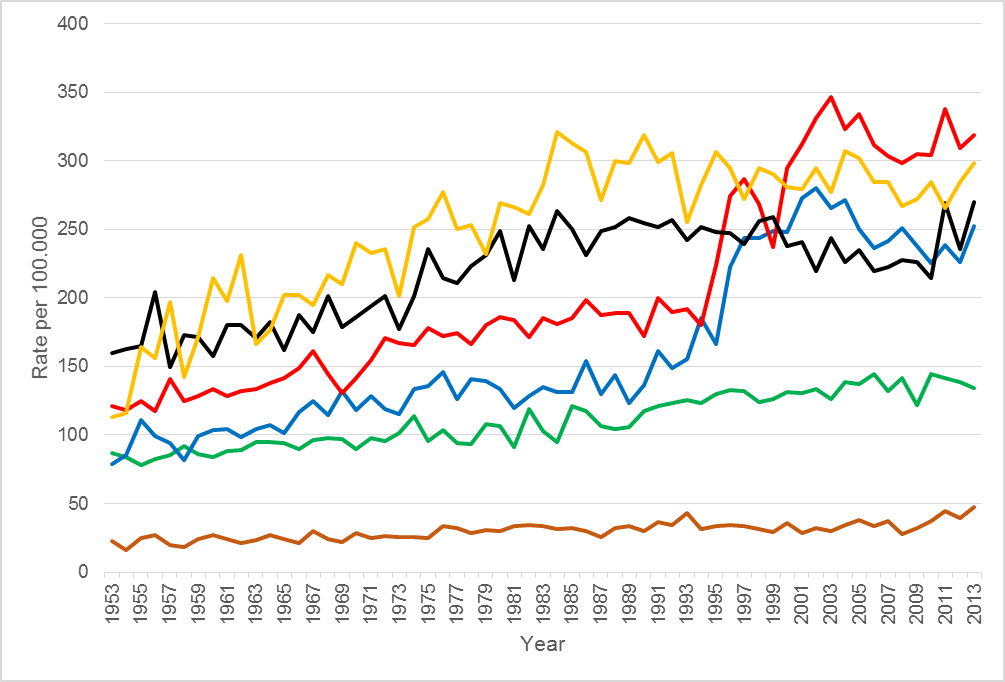


S10. Observed female breast cancer incidence in Norway from 1953 – 2013. Brown = age 30-39, green = age 40-49, blue = age 50-59, red = age 60-69, black = age 70-79, orange = age 80+.

Source: NORDCAN-database


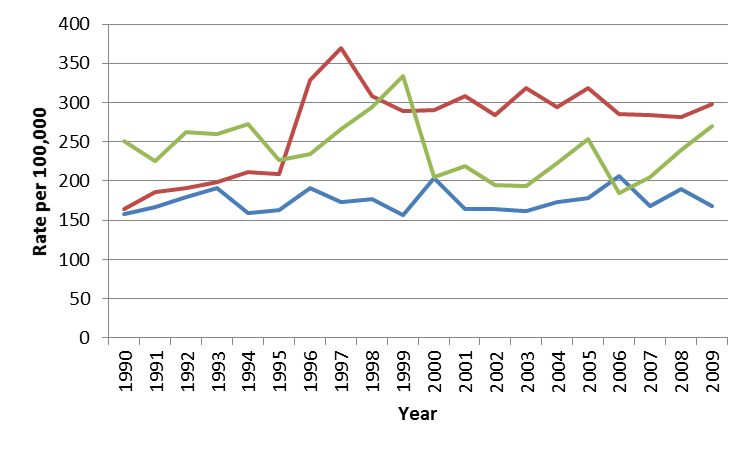


Figure S11: Observed incidence in the AORH-counties from 1990-2009. Blue = age 45-49, Red = age 50-69 and green = age 70-74.

1. **Dormant tumors**


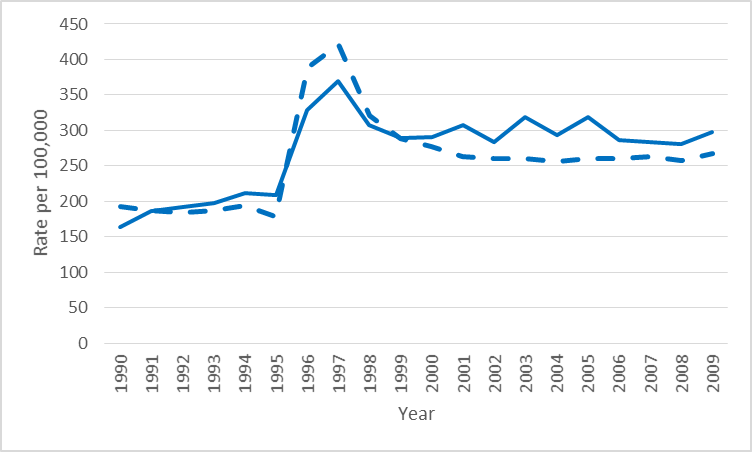


Figure S12: Simulation results with dormant tumors which are not detected clinically. Solid line = Observed incidence, dashed line = simulated incidence.

1. **Effect of opportunistic screening**

**
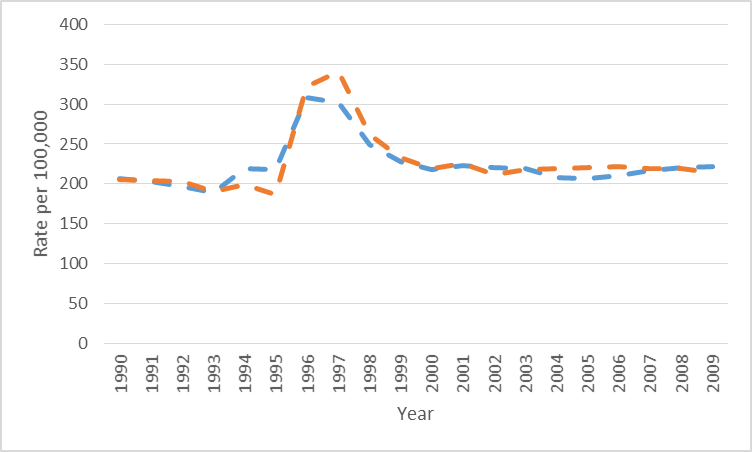
**

Figure S13: Effect of adding two hypothetical rounds of opportunistic screening (1992-1993 and 1994-1995), where 20% attend the first and 40% attend the second. Orange = no opportunistic screening, Blue = opportunistic screening.
